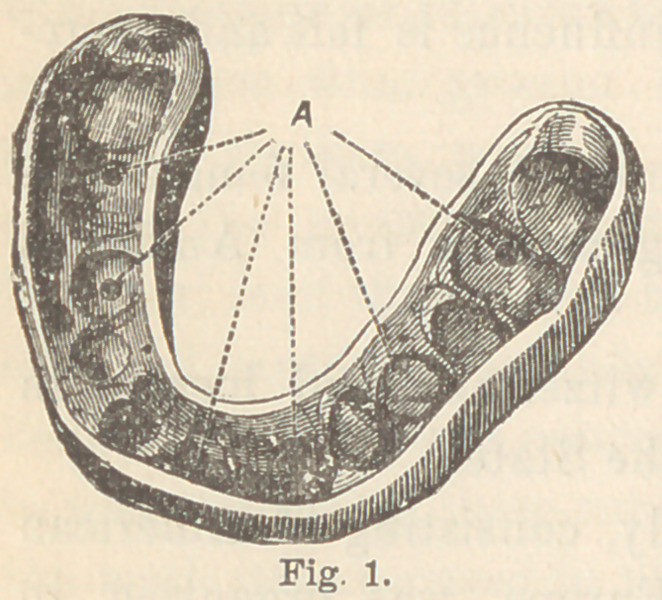# A Case of Fracture of the Inferior Jaw

**Published:** 1884-02

**Authors:** T. B. Gunning

**Affiliations:** New York


### A CASE OF FRACTURE OF THE INFERIOR JAW.

BY DR. T. B. GUNNING, NEW YORK.

Wm. McM., forty-two years old, was, for the purpose of robbery beaten insensible, and his lower jaw broken between the left incisor teeth. A large cut behind the chin extended up to the floor of the mouth. The face generally was much swollen, with great pain in the glenoid cavities and around the condyles. The displacement of the fragments was very determined, and bandages applied by the medical practitioners were unbearable. Subsequently, at the New York Hospital, the teeth were wired together, but this failing to hold the fragments in place, it was decided to apply a hard rubber splint.

The lower jaw contained sixteen teeth, which were originally short, and by wear were by this time unusually so. A wax impression was taken of the whole at once ; the plaster cast from this was separated at the point of fracture, and the parts set in place with the assistance of a cast of the upper teeth, on the plan first made known by Mr. John Tomes. On putting the splint on (See fig. 1), twelve days after the injury, some difficulty was experienced in getting the teeth into place, through adhesions holding the fragments of the jaw in displacement. Several openings were cut in the splint, one, the largest, in front of the teeth, each side of the fracture, in order to judge the positions of the fragments of the jaw. Although the jaw was allowed its natural movement, the splint was not fastened to the teeth in any way whatsoever, the broken jaw being left to the control of its muscles, which, especially the elevators in closing, are a counter-support to the splint, and force the teeth and jaw upward. In the fourth week after the application of the splint three pieces of necrosed bone were removed with forceps, and in a few days afterward another was removed from the mouth of the wound with the fingers. The discharge of pus was much lessened by this, and in five weeks from the application of the splint the wound beneath the chin was nearly closed. When the splint was removed in the second week from its application, there was but little displacement of the fragments, and on the thirty-fifth day after its application there was good union between the fragments, although it was necessarily very soft, through the proximity of the suppurating wound. From the time the splint was applied the pain rapidly disappeared, and the man has been quite comfortable except from the pain of the wound when touched. Now that this is healed, there is every probability that the union of the fracture will rapidly ossify.

Fig. 1.

The jaw was broken on September 17th, and the splint applied on the 29th, twelve days after. It was taken off frequently during the first thirty days, in the first instance to show the fracture to Dr. Weir's surgical class in the College of Physicians and Surgeons, and afterwards to remove the necrosed bone before spoken of. A report was first made to the New York Clinical Society, but this ended with the weekly observation of the case on Saturday, November 3rd, at which time it was supposed that the hole under the chin would be virtually closed in a few days. It is, however, still discharging, although no fragments of bone have been removed since those in October. The swelling on the inside of the mouth is nearly gone, and the loosened incisors are growing firm, while the hardening of the union is progressing satisfactorily.

The premature meeting of the lower back teeth with the upper ones, is, perhaps, owing to injury to the capsular ligaments which cover the condyles, but no apprehension need be felt but that the front teeth will close firmly in three or four months. The chief importance of this case lies in the fact that it is a complete demonstration that an inter-dental splint which covers all the teeth of the lower jaw, will hold the fragments in place when the fracture is between the canine teeth, without external appliances. In fact it will do this in nearly all cases, even when the fracture is as far back as the first permanent molar. This important feature was first demonstrated in a patient in the Bellevue Hospital, whose jaw was broken in December, 1863. See N. Y. Med. Jour., vol. iv., p. 14.

## Figures and Tables

**Fig. 1. f1:**